# Involvement of the SATB1/F-actin complex in chromatin reorganization during active cell death

**DOI:** 10.3892/ijmm.2014.1710

**Published:** 2014-03-21

**Authors:** DARIUSZ GRZANKA, MACIEJ GAGAT, MAGDALENA IZDEBSKA

**Affiliations:** 1 Department and Clinic of Dermatology, Sexually Transmitted Diseases and Immunodermatology, Nicolaus Copernicus University in Toruń, Collegium Medicum in Bydgoszcz, 85-092 Bydgoszcz, Poland; 2 Department of Histology and Embryology, Nicolaus Copernicus University in Toruń, Collegium Medicum in Bydgoszcz, 85-092 Bydgoszcz, Poland

**Keywords:** F-actin, sequence-binding protein 1, active cell death, confocal microscopy, transmission electron microscopy

## Abstract

Over the past years, confirmations on the presence of actin and/or its polymerized form, F-actin, in the cell nucleus are progressively accumulating. Nevertheless, the function and localization of F-actin in the nucleus is still not fully characterized. Thus, the aim of the present study was to evaluate the association between F-actin and sequence-binding protein 1 (SATB1) and their involvement in chromatin remodeling associated with active cell death. Both SATB1 and F-actin were colocalized in the transcriptional active regions of the cell nucleus and a functional interaction was observed between SATB1 and higher-organized nuclear F-actin structures at the border between condensed and decondensed chromatin. These results extend the knowledge on the role of SATB1 and nuclear F-actin in three-dimensional chromatin organization and their functions during active cell death. Additionally, this study opens the discussion on the involvement of the SATB1/F-actin functional complex in active cell death; further studies are required to fully elucidate these issues.

## Introduction

It has long been suggested that nuclear actin exists only in a globular form as G-actin (1,2). However, increasing evidence demonstrates the nuclear localization of F-actin as part of a large structure in the nucleoplasm and indicates that it interacts with nuclear matrix proteins (3–8). The first studies that reported on the presence of nuclear actin, or its polymerized form, were treated with skepticism and this issue has been questioned for decades (9). The main problem occurs in detecting the short polymers of F-actin in the nucleus by fluorescence staining or at the ultrastructural level. However, newly developed methods and technological progress allow us to shed new light on the localization and involvement of actin or its polymerized form in many cellular compartments. The difficult detection of nuclear actin filaments is caused by their size (3–5 μm in diameter) and by the fact that nuclear actin cannot be stained with phalloidin (10). In the present study, we used both confocal and transmission electron microscopy (TEM) to detect actin filaments in the cell nucleus. As previousy demonstrated Izdebska *et al*, the combination of pre- and post-embedding techniques is important in the phalloidin-based detection of nuclear F-actin by TEM. Moreover, they suggested that the use of streptavidin-coated quantum dots (QDs), through the different strategy of their bioconjugation, eliminates weak labeling problems (11). Nevertheless, the function and localization of actin in the nucleus is not yet fully characterized. Undoubtedly, actin exists in various forms and is involved in distinct functions in the nucleus. It is also known to play an essential role in the nuclear architecture and functions (7,12,13). Additionally, actin is localized in the nucleoli, splicing speckles and Cajal bodies (14–16). It has been suggested that in these nuclear compartments, actin is associated with gene expression as a part of chromatin remodeling complexes, linked with the transcription machineries, as well as with mRNA nuclear export (17–21). Other nuclear processes in which actin is involved include the assembly of the nuclear structure genome organization, and the regulation of transcription factor activity (17,22–24). Hence, actin seems to be a crucial protein which interacts with nuclear components, particularly with matrix proteins and chromatin.

Zimmer and Fabre indicated that spatial chromatin organization within the highly organized and dynamic structure as the cell nucleus affects genome expression, stability and replication (25). As it has been suggested by Spilianakis *et al*, it is also possible that genes on different chromosomes cooperate in a coordinated manner, thus altering their expression at the same time (26). Moreover, spatial chromatin organization is directly influenced by the interactions of chromatin and nuclear matrix proteins accordingly, to control gene transcription and DNA repair mechanisms (27). One of these identified matrix proteins is special AT-rich sequence-binding protein 1 (SATB1) that binds specifically to AT-rich sequences of DNA by recognizing the base unpairing regions (BURs) (28). The SATB1 protein may coordinate the control of the expression of various genes even in distant parts of the genome through the regulation of chromatin-loop architecture and transcription. It forms three-dimensional functional cage-like structures inside the cell nucleus which anchor chromosomes by tethering the BUR regions of the selected gens (29). SATB1 seems to be of particular importance to cells which must alter their functions.

The role of SATB1 in the regulation of apoptotic cell death process has also been reported (30–33). On the one hand, these studies were based on the degradation of SATB1 in cells undergoing apoptosis (31,32,34). On the other hand, there is no study which describes the link between theapoptotic process and the high level of SATB1 expression (32). Moreover, to our knowledge, in the literature, there is no information on its expression during mitotic catastrophe.

Due to the evidence that the SATB1 protein provides the structural background for chromatin organization, it can be significant in the reorganization of chromatin during cell death. It is also possible that SATB1 and nuclear actin or nuclear F-actin may jointly act to promote changes in chromatin and nuclear architecture associated with active cell death process. It has been demonstrated by a number of authors that nuclear actin is involved in chromatin remodeling in various nuclear pathways, such as transcription, RNA export and intra-nuclear transport (7,17,35–40).

In our previous studies, we have also provided evidence of the polymeric forms of actin in the nucleus of different cell lines following the induction of active cell death processes (11,41–44). Continuing with our findings, the aim of the present study was to evaluate the association between nuclear F-actin and SATB1 in chromatin remodeling associated with active cell death and by this means to reveal the functional association between these two nuclear matrix proteins. These results extend the knowledge on the role of SATB1 and nuclear actin in three-dimensional chromatin organization and its influence on active cell death.

## Materials and methods

### Cell culture and treatment

The Chinese hamster ovary (CHO AA8) cell line, which was kindly provided by Professor M.Z. Zdzienicka (Department of Molecular Cell Genetics, Nicolaus Copernicus University in Toruń, The Ludwik Rydygier Collegium Medicum in Bydgoszcz, Poland), was cultured in Eagle’s minimum essential medium (MEM; Sigma-Aldrich, St. Louis, MO, USA) supplemented with 10% fetal bovine serum (Gibco, Grand Island, NY, USA) and 50 μg/ml gentamycin (Sigma-Aldrich) in 5% CO_2_ at 37°C. For the induction of cell death, CHO AA8 cells were grown in six-well plates (Falcon; BD Biosciences, San Jose, CA, USA) and treated with doxorubicin (Sigma-Aldrich) at a concentration of 2.5 μM for 24 h. The control cells were grown under the same conditions without doxorubicin treatment.

### Cell death analysis

The analysis of cell death was performed using a Tali Image-based cytometer and a Tali Apoprosis kit (both from Invitrogen Life Technologies, Carlsbad, CA, USA) according to the manufacturer’s instructions. Briefly, following trypsinization, the suspended cells were resuspended in Annexin binding buffer at a concentration of approximately 5×10^5^ to 5×10^6^. Subsequently, to each 100 μl of sample, 5 μl of Annexin V Alexa Fluor 488 were added, mixed and incubated at room temperature in the dark for 20 min. The cells were then centrifuged and resuspended in 100 μl of Annexin binding buffer. After the addition of 1 μl of propidium iodide (PI) to each sample, the cells were incubated at room temperature in the dark for 3 min. A total of 25 μl of stained cells were loaded into a Tali Cellular Analysis Slide (Invitrogen Life Technologies). The data were analyzed using FCS Express Research Edition software (Ver4.03; De Novo Software, Los Angeles, CA, USA) on the assumption that viable cells are both Annexin V Alexa Fluor 488- and PI-negative cells, cells that are in early apoptosis are Annexin V Alexa Fluor 488-positive and PI-negative, cells that are in late apoptosis are both Annexin V Alexa Fluor 488- and PI-positive, whereas necrotic cells are Annexin V Alexa Fluor 488-negative and PI-positive.

### Fluorescence localization of F-actin and SATB1

For the fluorescence localization of F-actin and SATB1, the cells grown on sterile glass coverslips were fixed in 4% (w/v) paraformaldehyde in PBS, pH 7.4 (20 min, room temperature). After rinsing with PBS (3×5 min), permeabilization using 0.1% (v/v) Triton X-100 (Serva Electrophoresis GmbH, Heidelberg, Germany) and washing with PBS (3×5 min), the non-specific protein interactions were blocked with 1% (w/v) BSA/PBS (Sigma-Aldrich) for 30 min. Subsequently, SATB1 was labeled by incubating the cells with primary antibodies diluted in blocking buffer for 1 h at room temperature [rabbit monoclonal anti-SATB1 (Abcam Cambridge, MA, USA) or rabbit polyclonal anti-SATB1 (1:50; Sigma-Aldrich)]. After washing (3X PBS), the cells were incubated with a secondary antibody [Alexa Fluor 488 goat anti-rabbit IgG (H+L; Invitrogen Life Technologies)] for 1 h at room temperature, in the dark. For the localization of F-actin, the cells were incubated with phalloidin-TRITC conjugate (1:5; Sigma-Aldrich) or Alexa Fluor 488-phalloidin (1:20; Invitrogen Life Technologies) for 20 min in the dark. After washing (3X PBS), the cell nuclei were counterstained with DAPI (1:20,000; Sigma-Aldrich). The slides were mounted in Aqua-Poly/Mount (Polysciences, Inc., Warrington, PA, USA) and examined using C1 laser-scanning confocal microscope (Nikon, Tokyo, Japan) with an (×100) oil immersion objective. Images were captured using Nikon EZ-C1 software (Ver3.80; Nikon).

### 5′-Fluorouridine (5-FUrd) incorporation and its colocalization with F-actin or SATB1

For 5-FUrd incorporation, the cells grown on sterile glass coverslips were incubated in complete medium containing 5-FUrd for 20 min at 37°C in 5% CO_2_ and then fixed in 4% (w/v) paraformaldehyde in PBS, pH 7.4 (20 min, room temperature). After rinsing with PBS (3×5 min), permeabilization using 0.1% (v/v) Triton X-100 (Serva Electrophoresis GmbH) and washing with PBS (3×5 min), the non-specific antibody interactions were blocked with 1% (w/v) BSA/PBS (Sigma-Aldrich) for 30 min. Subsequently, 5′-FUrd was labeled by incubating the cells with primary mouse monoclonal anti-BrdU (Sigma-Aldrich) antibody diluted 1:250 in blocking buffer for 1 h, at room temperature [mouse monoclonal anti-BrdU (Sigma-Aldrich)]. After washing (3X PBS), the cells were incubated with Alexa Fluor 555 goat anti-mouse IgG (H+L; 1:500; Invitrogen Life Technologies) for 1 h at room temperature in the dark. After washing with PBS (3×5 min), F-actin or SATB1 were localized as described above. After washing (3X PBS), cell nuclei were counterstained with DAPI (1:20,000; Sigma-Aldrich). The slides were mounted in Aqua-Poly/Mount (Polysciences) and examined using a C1 laser-scanning confocal microscope (Nikon) with an (×100) oil immersion objective. Images were captured using Nikon EZ-C1 software (Ver3.80; Nikon).

### Analysis of colocalization between SATB1/F-actin, F-actin/5-FUrd, and SATB1/5-FUrd

The analysis of the degree of the overlap between the two channels in the fluorescence images was performed using imageJ software (Ver1.47i) and the Colocalization Threshold plugin. Data are presented as a colocalization pixel map and a 2D intensity histogram with indicated linear regression.

### Ultrastructural colocalization of F-actin and SATB1

For the ultrastructural colocalization of SATB1, a post-embedding immunogold method was used. The harvested cells were fixed in 4% (w/v) paraformaldehyde in PBS, pH 7.4 (20 min, room temperature) and embedded in LR White. The immune-detection of SATB1 was performed on ultra-thin sections of study material collected on nickel grids by 1 h of incubation at room temperature with primary antibodies [anti-SATB1 antibody (1:50; Sigma-Aldrich)]. After washing in PBS containing 1% (w/v) BSA, the sections were incubated for 1 h with secondary antibodies tagged with 5 or 20 nm gold particles [donkey-anti-rabbit IgG (Aurion, Toowong, Australia)]. In the negative control, the primary antibody was replaced with PBS containing 1% (w/v) BSA. Following immune detection, the ultrathin sections were stained with uranyl acetate and lead citrate and examined under a JEM 100 CX electron microscope (Jeol, Ltd., Tokyo, Japan) operating at 80 kV.

For the ultrastructural localization of F-actin, the phalloidin-based method with semiconductor Zn/Se nanocrystals was used as previously described (44). Briefly, the harvested cells were fixed in 4% (w/v) paraformaldehyde in PBS, pH 7.4 (20 min, room temperature). After rinsing with PBS (3×5 min), permeabilization using 0.1% (v/v) Triton X-100 (Serva Electrophoresis GmbH) and washing with PBS (3×5 min), the non-specific protein interactions were blocked with 6% (w/v) BSA/PBS (Sigma-Aldrich) for 1 h. Subsequently, F-actin was labeled by incubation of the study material with biotinyled phalloidin (1:85; Sigma-Aldrich) diluted in blocking buffer for 20 min. In the next step, after rinsing with PBS (3×5 min), the study material was post-fixed in 1% (w/v) OsO_4_ (Serva Electrophoresis GmbH), dehydrated with a graded series of ethanol, infiltrated and embedded in LR White. Ultrathin sections were placed on nickel grids (Sigma-Aldrich). The detection of biotinyled phalloidin (Sigma-Aldrich) was performed by using Qdot^®^ 525 streptavidin conjugate (1:100; Invitrogen Life Technologies) diluted in blocking buffer for 1 h. After staining with uranyl acetate, the preparations were examined under a JEM 100 CX electron microscope (Jeol, Ltd.) operating at 80 kV.

### FRET acceptor bleaching (AB) method

For the SATB1 interactions with F-actin, the FRET AB method was used. The images were collected using a C1 laser-scanning confocal microscope (Nikon) equipped with three lasers: i) diode 405 nm, ii) diode 488 nm and iii) He-Ne 543 nm. Images were captured using Nikon EZ-C1 software (Ver3.80; Nikon). The preparations for FRET analysis were carried out analogically as for the immunofluorescence experiments, but DAPI counterstaining was omitted. Briefly, F-actin labeling was performed using phalloidin-TRITC conjugate (Sigma-Aldrich) and as the secondary antibody for SATB1 labeling, Alexa Fluor 488 goat anti-rabbit IgG (H+L; Invitrogen Life Technologies) was used. Alexa Fluor 488 (acceptor) was induced using a diode laser 488 nm, while TRITC (donor) was induced using a HeNe laser 543 nm. FRET AB method was performed by comparing donor fluorescence intensity in the same sample before and after destroying the acceptor by photobleaching. If FRET was initially present, a resultant increase in donor fluorescence would occur on photobleaching of the acceptor. The energy transfer efficiency was calculated using the following formula: FRETeff = (Dpost-Dpre)/Dpost; where Dpost is the fluorescence intensity of the Donor after acceptor photobleaching and Dpre the fluorescence intensity of the Donor before acceptor photobleaching. The FRET efficiency was considered as positive when Dpost > Dpre.

### Co-immunoprecipitation

For the analysis of the presence of SATB1 in the protein complex with β-actin, the Direct IP kit (Pierce Thermo Scientific, Rockford, IL, USA) was used according to the manufacturer’s instructions. Briefly, the cells were lysed using IP Lysis buffer (Pierce Thermo Scientific). After normalization of the protein concentration by the BCA Protein Assay kit (Pierce Thermo Scientific) using a spectrophotometer, equal amounts of protein were incubated with 150 μg antibody against β-actin. Subsequently, the lysates were incubated with protein A/G Plus beads (Pierce Thermo Scientific). Proteins were analyzed by western blot analysis.

### Pull-down assay

For the analysis of the presence of SATB1 in the protein complex with F-actin, streptavidin-coated magnetic beads (Dynabeads M-280 Streptavidin; Invitrogen Life Technologies) were used according to the manufacturer’s instructions with a number of key modifications. Briefly, the cells were lysed in RIPA buffer in the presence of biotinyled phalloidin (1:85) (both from Sigma-Aldrich). Following lysate clarification by centrifugation at 8 000 × g for 10 min at 4°C, each lysate was divided into two fractions: ‘supernatant’ and ‘debris’. Following the normalization of protein concentration in ‘supernatant’ by the BCA Protein Assay kit (Pierce Thermo Scientific) using a spectrophotometer, equal amounts of protein were incubated with 0.5 mg of streptavidin-coated magnetic beads for 30 min at room temperature with gentle rotation of the tube. ‘Debris’ was resuspended in RIPA buffer supplemented with biotinyled phalloidin (1:85) and analogically incubated with streptavidin-coated magnetic beads. After five cycles of magnetic separation/washing in PBS containing 0.1% BSA, the biotin-streptavidin bonds were broken by boiling the samples for 5 min in 0.1% SDS. Proteins were analyzed by western blot analysis.

### Western blot analysis

Semi-quantitative analysis of the post-translational expression of SATB1 or the detection of SATB1 in the protein complex with β-actin or F-actin was performed by western blot analysis. Following the normalization of the protein concentration by the BCA Protein Assay kit (Pierce Thermo Scientific) using a spectrophotometer, equal amounts of protein (18 μg of total protein per lane) were separated by 4–12% NuPage Bis-Tris gel (Novex; Invitrogen Life Technologies) and transferred onto nitrocellulose membranes using the iBlot dry western blotting system (Invitrogen Life Technologies). Following co-immunoprecipitation and pull-down, 18 μl of diluted protein complexes were separated. Pre-stained molecular weight markers (Pierce Thermo Scientific) were used to estimate the position of the protein bands. Subsequently, the membranes were processed using a BenchPro 4100 card processing station (Invitrogen Life Technologies). The membranes were blocked with 5% non-fat milk in TBS-T for 2 h and then incubated with primary rabbit monoclonal anti-SATB1 (1:100; Abcam), rabbit polyclonal anti-SATB1 (1:250) and rabbit anti-GADPH (1:5,000) (both from Sigma-Aldrich) antibodies diluted TBS-T for 1 h at room temperature. After washing with TBS-T, the membranes were incubated with secondary goat anti-rabbit antibodies conjugated with alkaline phosphatase (1:30,000; Sigma-Aldrich) diluted in TBS-T for 1 h at room temperature. Protein bands were visualized using NBT/BCIP ready-to-use tablets (Roche Applied Science, Indianapolis, IN, USA).

### Statistical analysis

The data are presented as the means ± SEM. Statistical comparisons between two groups of cell death or fluorescence intensity data were performed using a two-tailed Mann-Whitney U test. The differences between the groups were considered significant at P<0.05. The GraphPad Prism 5.0 (GraphPad Software) was used for statistical analyses.

## Results

### Doxorubicin induces active cell death

**Figure 1:**
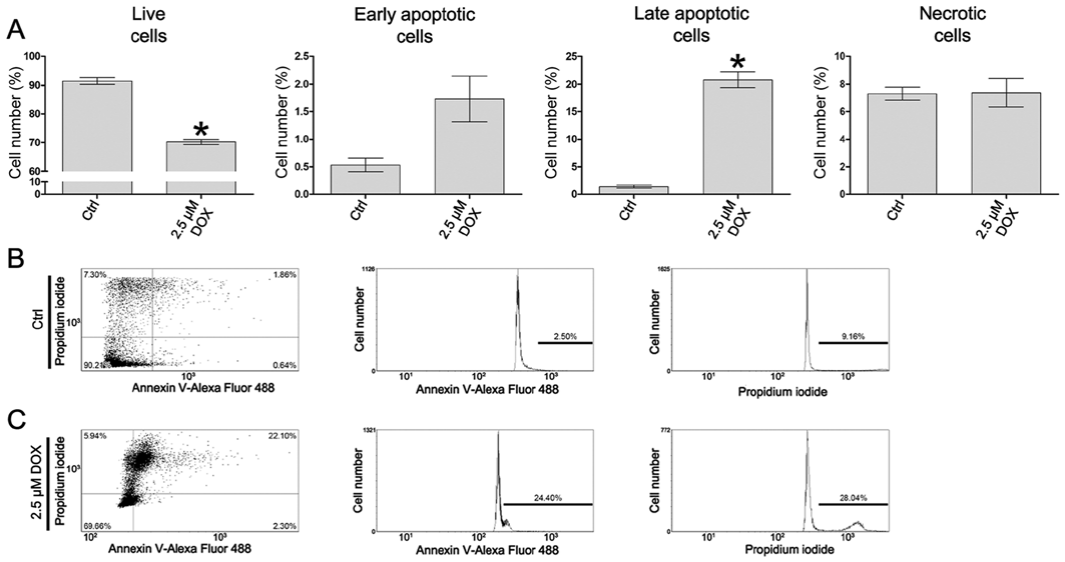
Effect of doxorubicin on the induction of cell death. CHO AA8 cells were treated with 2.5 μM doxorubicin for 24 h and double-stained with Annexin V Alexa Fluor 488 and propidium iodide (PI). Analysis was performed using an image-based cytometer. (A) Percentage of live, early apoptotic, late apoptotic and necrotic cells. ^*^ P<0.05, statistically significant differences, as shown by Mann-Whitney U test. (B) Representative histogram of Annexin V Alexa Fluor 488-positive cells in the control. (C) Representative histogram of PI-positive cells following treatment with 2.5 μM doxorubicin. Ctrl, control; DOX, doxorubicin.

The analysis of cell death was performed using a Tali Image-based cytometer following Annexin V Alexa Fluor 488 and PI double staining. In the control cells, the mean percentage of Annexin V-positive cells was 1.96%, whereas the mean percentage of PI-positive cells was 7.30%. Following treatment of the cells with 2.5 μM doxorubicin, the mean percentage of Annexin V-positive cells was 22.49%, whereas the mean percentage of PI-positive cells was 7.37%. Based on the assumption described in ‘Material and methods’, in the CHO AA8 cells treated with 2.5 μM doxorubicin, a statistically significant decrease in the percentage of live cells was observed, as compared to the control (from 91.49 to 70.20%, P=0.0286). Additionally, following treatment of the cells with doxorubicin at the concentration of 2.5 μM, a statistically significant increase in the percentage of late apoptotic cells was observed (from 1.45 to 20.77%, P=0.0286). The differences between the populations of early apoptotic and necrotic cells following treatment with 2.5 μM doxorubicin in comparison to the controls were statistically insignificant (P=0.2000 and P=0.6631, respectively) (Fig. 1A–C).

### Doxorubicin treatment increases SATB1/F-actin colocalization

**Figure 2:**
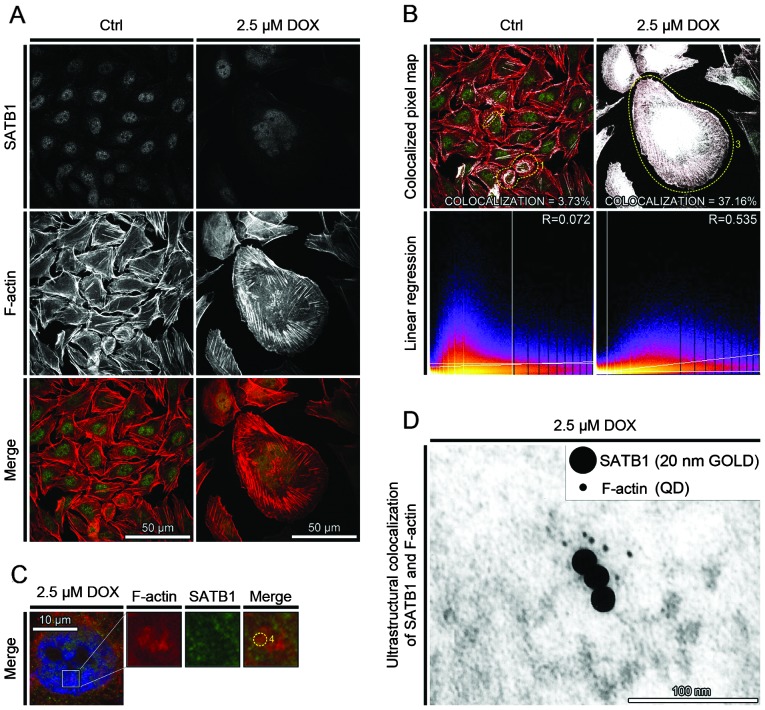
Colocalization of sequence-binding protein 1 (SATB1) and F-actin. CHO AA8 cells were treated with 2.5 μM doxorubicin for 24 h and labeled for SATB1 and F-actin. (A) Localization of SATB1 and F-actin in the control and cells treated with 2.5 μM doxorubicin. (B) Analysis of fluorescence colocalization of SATB1 and F-actin using a confocal microscope in the control cells and cells treated with 2.5 μM doxorubicin. (C) Fluorescence colocalization of SATB1 and F-actin in the area of nucleus of cell treated with 2.5 μM doxorubicin. Numbered dotted cirles indicate: 1, colocalization of SATB1 and thick F-actin stress fiber; 2, colocalization of SATB1 and F-actin in dividing cells; 3, colocalization of SATB1 and F-actin in cells undergoing active cell death; 4, colocalization of SATB1 and F-actin in the weak DNA labeling area of the nucleus of cells undergoing active cell death. (D) Ultrastructural colocalization of SATB1 and F-actin in the area of cell nucleus of cell treated with 2.5 μM doxorubicin. Ctrl, control, DOX, doxorubicin.

The fluorescence analysis of SATB1 localization in the control cells revealed its presence in the area of the cell nucleus in the form of foci and it was scattered in the cytoplasm. Following the induction of cell death, SATB1 protein was localized mostly in the cell nucleus. In the cortical cytoplasm of the cells treated with 2.5 μM doxorubicin, SATB1 formed clearly visible filament-like structures, associated with F-actin stress fibers (Fig. 2A).

The analysis of the colocalization between SATB1 and F-actin indicated its increase after the induction of cell death. The percentage of colocalized pixels increased from 3.73% (R=0.072) to 37.16% (R=0.535) for the control cells and the cells treated with 2.5 μM doxorubicin, respectively. In the controls, the colocalization of SATB1 and F-actin was observed in association with actin polymers in freshly divided cells (Fig. 2B, dotted circle 1) or along a few thick tension cytoplasmic fibers of F-actin (Fig. 2B, dotted circle 1). Following treatment of the cells with 2.5 μM doxorubicin, SATB1 protein was colocalized with nuclear F-actin or was associated with F-actin cytoplasmic stress fibers (Fig. 2B, dotted circle 3). Moreover, in the cell nucleus, SATB1 was colocalized with more densely organized nuclear F-actin ring-like structures, at the area of weak DAPI staining (Fig. 2C, dotted circle 4).

Additionally, on the ultrathin sections of the CHO AA8 cells treated with 2.5 μM doxorubicin labeled for the presence of SATB1 (20 nm gold) and F-actin (Qdots 525), the double-labeling was distributed throughout the nucleoplasm. The complexes of SATB1 and F-actin were localized at the border of electron-dense regions of the cell nuclei, possibly regions between condensed and decondensed chromatin (Fig. 2D).

### Doxorubicin increases the colocalization of both SATB1/5FUrd and F-actin/5FUrd

To investigate whether SATB1 or F-actin have functional consequences in transcriptional processes during active cell death, the colocalization of the above mentioned proteins with 5-FUrd incorporation into nascent mRNA was performed.

**Figure 3:**
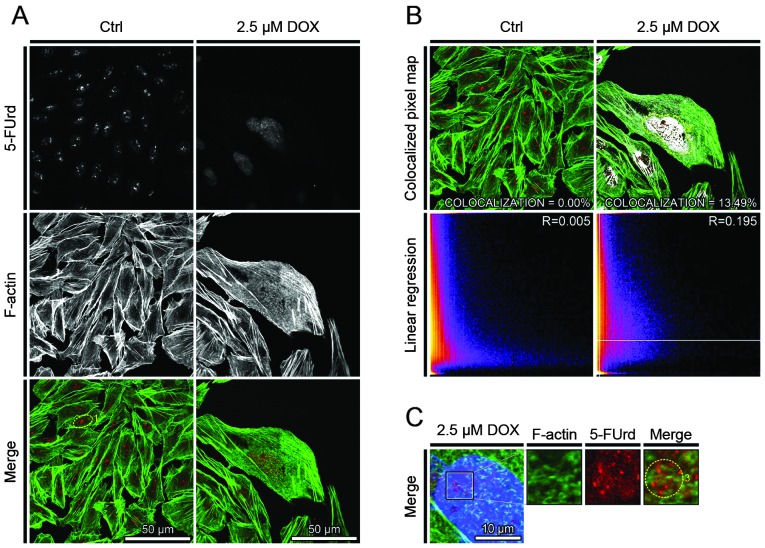
Colocalization of F-actin and 5′-fluorouridine (5-FUrd). CHO AA8 cells were treated with 2.5 μM doxorubicin for 24 h, incubated with 5-FUrd for 20 min and labeled for both 5-FUrd and F-actin. (A) Localization of F-actin and 5-FUrd in the control and cells treated with 2.5 μM doxorubicin. (B) Analysis of fluorescence colocalization of F-actin and 5-FUrd using a confocal microscope in the control cells and cells treated with 2.5 μM doxorubicin. (C) Fluorescence colocalization of F-actin and 5-FUrd in the area of the nucleus of cells treated with 2.5 μM doxorubicin. Numbered dotted circles indicate: 1, the lack of colocalization of 5-FUrd and F-actin; 2, colocalization of 5-FUrd and F-actin in the area of the nucleus of cells undergoing active cell death; 3, colocalization of 5-FUrd and F-actin in the weak DNA labeling area of the nucleus of cells undergoing active cell death. Ctrl, control; DOX, doxorubicin.

**Figure 4:**
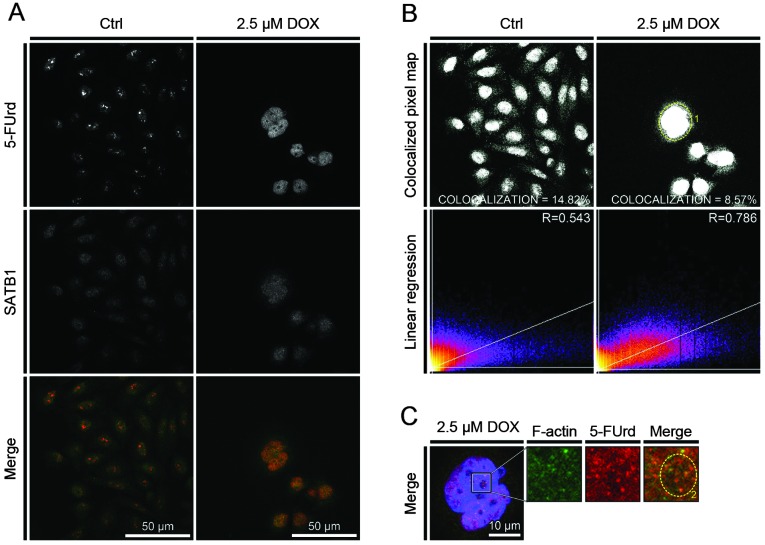
Colocalization of sequence-binding protein 1 (SATB1) and 5′-fluorouridine (5-FUrd). CHO AA8 cells were treated with 2.5 μM doxorubicin for 24 h, incubated with 5-FUrd for 20 min and labeled for both 5-FUrd and SATB1. (A) Localization of SATB1 and 5-FUrd in the control cells and cells treated with 2.5 μM doxorubicin. (B) Analysis of fluorescence colocalization of SATB1 and 5-FUrd using a confocal microscope in the control cells and cells treated with 2.5 μM doxorubicin. (C) Fluorescence colocalization of SATB1 and 5-FUrd in the area of nucleus of cells treated with 2.5 μM doxorubicin. Numbered dotted cirles indicate: 1, colocalization of 5-FUrd and SATB1 in the area of the nucleus of cells undergoing active cell death; 2, colocalization of 5-FUrd and SATB1 in the weak DNA labeling area of the nucleus of cells undergoing active cell death. Ctrl, control; DOX, doxorubicin.

In the controls, 5-FUrd 2–4 large foci, with high fluorescence intensity (Figs. 3A, dotted circle 1; and Fig. 4A) and small foci in the area of the cell nucleus were observed. Following treatment of the cells with 2.5 μM doxorubicin, the number of 5-FUrd small foci localized in the area of the cell nucleus increased. Moreover, the overall fluorescence intensity of detected 5-FUrd foci was higher in the cells with the phenotype of mitotic catastrophe, as compared with the controls (Figs. 3A and 4A).

The analysis of the colocalization between 5-FUrd and F-actin indicated its absence in the control cells and its increase after the induction of cell death. The percentage of colocalized pixels increased from 0.00% (R=0.005) to 13.49% (R=0.195) for the controls and the cells treated with 2.5 μM doxorubicin, respectively. Following treatment of the cells with 2.5 μM doxorubicin, 5-FUrd protein was colocalized with nuclear F-actin (Fig. 3B, dotted circle 2). Moreover, 5-FUrd was colocalized with nuclear F-actin ring-like structures, at the area of weak DAPI staining (Fig. 3C, dotted circle 3).

The analysis of the colocalization between 5-FUrd and SATB1 indicated its decrease following the induction of cell death. The percentage of colocalized pixels decreased from 14.82 (R=0.543) to 8.57% (R=0.786) for the controls and the cells treated with 2.5 μM doxorubicin, respectively (Fig. 4B). However, in the cells with the phenotype of mitotic catastrophe, the nuclear colocalization of 5-FUrd and SATB1 was characterized with a higher regression, indicating wide-genome transcriptional processes (Fig. 4B, dotted circle 1). Moreover, the colocalization of 5-FUrd and SATB1 was observed in both the regions of the cell nucleus stained weakly and intensively with DAPI (Fig. 4C, dotted circle 2).

### Doxorubicin treatment induces SATB1/F-actin interactions

**Figure 5:**
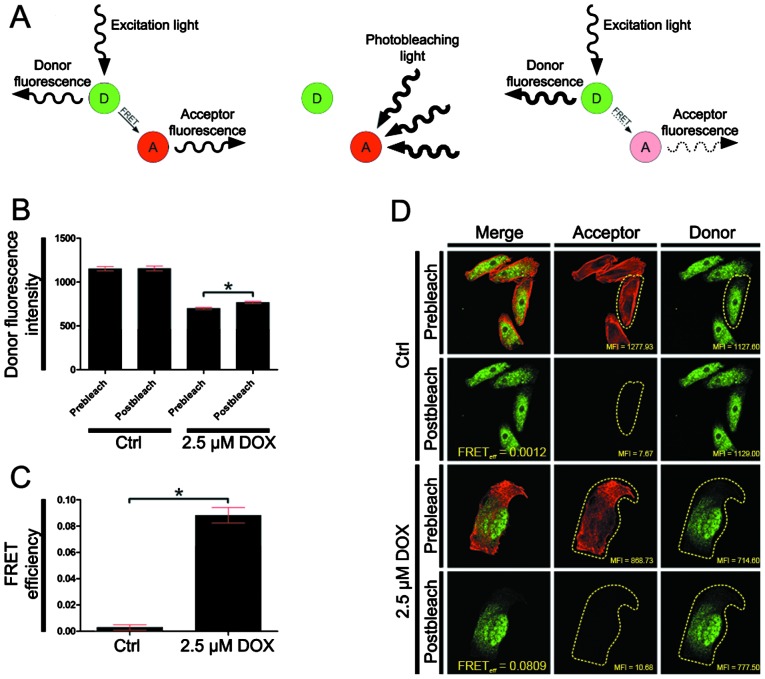
Analysis of sequence-binding protein 1 (SATB1)/F-actin interactions. (A) Schematic diagram of the FRET acceptor bleaching experiment. (B) Donor fluorescence intensity in the cells after acceptor photobleaching. (C) FRET efficiency analysis of SATB1/F-actin interactions in the control cells and cells treated with 2.5 μM doxorubicin. (D) Representative confocal micrographs of FRET efficiency analysis of SATB1/F-actin interactions in the control cells and cells treated with 2.5 μM doxorubicin. Ctrl, control; DOX, doxorubicin.

The fluorescence analysis of SATB1/F-actin interactions were investigated using the FRET acceptor bleaching method (Fig. 5A), in the control cells and cells undergoing mitotic catastrophe following treatment with 2.5 μM doxorubicin. In the control cells, the mean of donor fluorescence intensity was statistically insignificant and the energy transfer efficiency was 0.0029. This confirmed the examination of the fluorescence colocalization results and indicate the lack of SATB1/F-actin interactions. Following the induction of cell death, the mean of donor fluorescence intensity in the cells undergoing active cell death, was statistically significantly increased after acceptor photobleaching (from 700.20 to 767.91, P=0.0286, before and after acceptor photobleaching, respectively) and the mean energy transfer efficiency was 0.0881 (Fig. 5B–D). These data thus indicate that SATB1 interacts with F-actin and confirm the colocalizational resuts that indicate the functional association of both analyzed nuclear matrix proteins.

**Figure 6:**
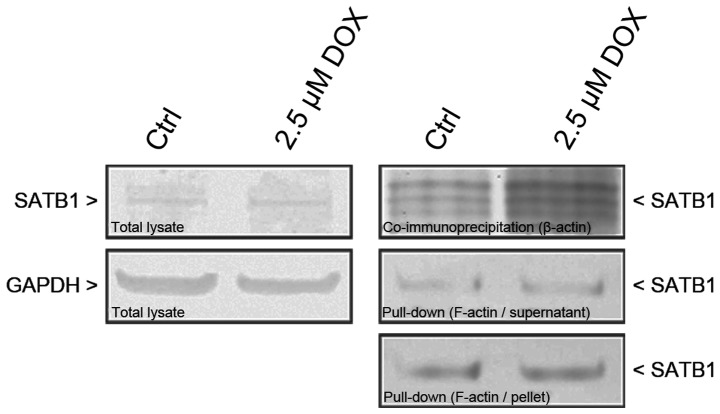
Analysis of β-actin/sequence-binding protein 1 (SATB1) or F-actin/SATB1 interactions using co-immunoprecipitation or pull-down assays. Ctrl, control; DOX, doxorubicin.

To investigate biochemically whether SATB1 interacts with actin *in vitro*, cell extracts from CHO AA8 fibroblasts were immunoprecipitated with an antibody to β-actin and subjected to the detection of SATB1 by western blot analysis. The results revealed significantly elevated levels of SATB1 in the immunoprecipitates obtained from the cells treated with 2.5 μM doxorubicin, and indicate that SATB1 interacts with β-actin more intensely following the induction of active cell death (Fig. 6). Consequently, to confirm fluorescence-based experiments, pull-down assay using phalloidin-biotin and streptavidin-coated magnetic beads was used to investigate whether SATB1 interacts with actin filaments. The results confirmed those already obtained and indicated the increased level of SATB1 following the induction of cell death. Moreover, pull-down assay revealed the interactions of SATB1 not only with F-actin short polymers present in the supernatant fractions, but also with more densely organized or membrane-associated actin filaments in the cell debris (pellet) (Fig. 6).

## Discussion

There are a number of reports linking nuclear actin with chromatin remodeling during several nuclear processes (7,17,35–40). Sjölinder *et al* demonstrated the association of nuclear actin, chromatin remodeling and RNA polymerase II transcription (36). Other studies have shown nuclear actin as a component of chromatin remodeling complexes and its involvement in the nuclear envelope assembly (17,22,23). It has been also listed that nuclear actin controls the transcription of its target genes through different mechanisms: i) specifically binding to a 27-nt repeat element in intron 4 of the endothelial nitric oxide synthase gene and regulating its expression (45,46); ii) participation in chromatin remodeling necessary for gene activation (47–49); iii) direct role in RNA transcription by being part of the pre-initiation complex with RNA polymerase II (39); or iv) participation in transcriptional elongation (50). Moreover, it has been speculated that under stress conditions, actin may translocate into the cell nucleus to function as a transcriptional modulator of gene transcription (51–53). In addition, numerous proteins which interact with G- and F-actin have been detected in the nucleus (2,54). Previously, we have provided evidence of F-actin presence in the nucleus of different cell lines treated with anti-cancer drugs and speculated that nuclear F-actin may be involved in chromatin remodeling processes during apoptosis and mitotic catastrophe (11,41–44). In this study, we confirmed the involvement of F-actin in nuclear processes accompanied with active cell death processes. Moreover, we demonstrate that the SATB1/F-actin complex is localized at the border of condensed and decondensed chromatin which suggests its involvement in the process of transcription. Fomproix and Percipalle postulated that the spatial restriction of actin filaments predominantly to the interchromatin space argues against their direct involvement of conventional actin filaments in transcription and chromatin remodeling (14). Moreover, Belin *et al (8) and* Dundr *et al* (55) indicated that nuclear actin filaments are too short to serve as tracks for the long-range transport of genetic loci cargo through the nucleus. The authors also referred to the lack of directed motion of nuclear actin filaments and the little or no colocalization between nuclear actin filaments and up to now identified nuclear myosins. However, the conformational differences and some post-translational modifications in nuclear actin may be responsible for its different polymerization (56–58).

The matrix associated regions of DNA [MAR-binding proteins (MARBPs)] are dynamic and their distribution is cell type- and cell cycle-dependent. Several MARPBs have been characterized as SATB1, SATB2, BRIGHT, Cux/CDP, Lamin A/B/C, HMG and SMAR1 (59–64). SATB1 is organized into a cage-like network anchoring loops of heterochromatin and tethering specialized DNA sequences and serves as a global platform for the assembly of chromatin remodeling or modifying complexes with the anchored genomic loci (65). It has been pointed that depending on its post-translational modifications, SATB1 activates or represses multiple genes (66). Furthermore, SATB1 forms a functional architecture within the cell nucleus, referred to as ‘the SATB1 network’ and functions as a regulatory network of gene expression (67–69). Although SATB1 function has been studied mostly using T cells, its expression in the nuclei of other cell types may exert global gene regulatory activities as well.

In the present study, SATB1 was colocalized with 5-FUrd in both the controls and cells with the phenotype of mitotic catastrophe, following treatment with doxorubicin. Furthermore, we observed a reduction in fluorescence intensity of both SATB1 and 5-FUrd. This is consistent with the results obtained in the study by Chu *et al,* namely that the downregulation of SATB1 expression is responsible for active cell death initiation (70).

While the decrease in fluorescence intensity of SATB1 and 5-FUrd was observed in this study, the colocalization analysis revealed an increased overlap of their fluorescence signal in the area of the cell nucleus. This enhances our knowledge on SATB1-mediated genome-wide transcription and indicates that during active cell death, SATB1 is involved in the transcription of a larger number of genes at the same time after the induction of active cell death, as opposed to cells grown under physiological conditions.

Similarly, the colocalization analysis of F-actin and 5-FUrd, performed in the present study, revealed an intense overlap of the nuclear fluorescence signal following the induction of active cell death. Our results are in agreement with the suggestions of Xu *et al*, that actin translocates into the cell nucleus to function as a transcriptional modulator under stress conditions and plays an important role in the regulation of gene transcription (51,52). Moreover, the comparison of the above mentioned results with the colocalizational analysis of SATB1 and F-actin, revealed the overlap of their fluorescence, particularly in the area of the nucleus of in the cells in which cell death was induced. Additionally, the analysis of the SATB1 and 5-FUrd fluorescence signal in the cell nucleus revealed their colocalization in more densely organized nuclear F-actin structures. These results correlate with the analysis of the colocalization of SATB1 and F-actin shown by TEM. Furthermore, both the FRET AB technique and phalloidin-based pull-down assay confirmed that the SATB1/F-actin complex exists. Taken together with the information that SATB1 was detected in β-actin immunoprecipited cell lysates, our results are in agreement with those of other studies, indicating that nuclear actin exists in multiple forms: monomeric, oligomeric and short-polymeric (3,19,71).

In conclusion, we observed the colocalization of SATB1 and F-actin in the transcriptional active regions of the cell nucleus and a functional interaction was observed between SATB1 and more densely organized nuclear F-actin structures at the border between condensed and decondensed chromatin. This contributes to the hypothesis that nuclear actin filaments are involved in chromatin remodeling associated with transcriptional processes during active cell death. Our data open the discussion on the involvement of the SATB1/F-actin functional complex in the architecture of the cell nucleus of cells undergoing active cell death and put forward several issues that need be resolved in future studies: i) the issue of which genes are transcribed by this complex; ii) the significance of the cooperation between F-actin and SATB1 in the regulation of active cell death processes; and iii) the role of F-actin in processes regulated by SATB1.
